# Notice

**Published:** 1883-11-20

**Authors:** 


### EDITORIALS AND MISCELLANEOUS.

**NOTICE.**—*Many of our subscribers have forgotten us in the matter of remitting their dues. Friends, please attend to this matter at once. We are obliged to have money to run the Journal. Our printers are Cash men.* *W.*

